# Hydration Patterns in Sodium Alginate Polymeric Matrix Tablets—The Result of Drug Substance Incorporation

**DOI:** 10.3390/ma14216531

**Published:** 2021-10-29

**Authors:** Ewelina Juszczyk, Piotr Kulinowski, Ewelina Baran, Artur Birczyński, Jolanta Klaja, Dorota Majda, Encarna Garcia-Montoya, Władysław P. Węglarz, Przemysław Dorożyński

**Affiliations:** 1 Research and Development Center, Celon Pharma S.A., Marymoncka 15, 05-152 Kazuń Nowy, Poland; ewelina.juszczyk@celonpharma.com; 2 Institute of Technology, The Pedagogical University of Kraków, Podchorążych 2, 30-084 Kraków, Poland; ewelina.baran@up.krakow.pl (E.B.); artur.birczynski@up.krakow.pl (A.B.); 3 Oil and Gas Institute—National Research Institute, Lubicz 25 A, 31-503 Kraków, Poland; klaja@inig.pl; 4 Faculty of Chemistry, Jagiellonian University, Gronostajowa 2, 30-387 Kraków, Poland; majda@chemia.uj.edu.pl; 5 Pharmaceutical Technology and Physico-Chemical Department, Universidad de Barcelona, Av. Joan XXIII 27-31, 08028 Barcelona, Spain; encarnagarcia@ub.edu; 6 Department of Magnetic Resonance Imaging, Institute of Nuclear Physics Polish Academy of Sciences, Radzikowskiego 152, 31-342 Kraków, Poland; wladyslaw.weglarz@ifj.edu.pl; 7 Department of Drug Technology and Pharmaceutical Biotechnology, Medical University of Warsaw, Banacha 1, 02-097 Warszawa, Poland; przemyslaw.dorozynski@wum.edu.pl

**Keywords:** hydrophilic matrix tablets, sustained release drug delivery, sodium alginate, polymer hydration, swelling, drug solubility, polymer dissolution, magnetic resonance imaging, mass transport, X-ray microtomography

## Abstract

The purpose was to show, using destructive/nondestructive methods, that the interplay between water, tablet structure, and composition determine the unique spatiotemporal hydration pattern of polymer-based matrices. The tablets containing a 1:1 *w*/*w* mixture of sodium alginate with salicylic acid (ALG/SA) or sodium salicylate (ALG/SNA) were studied using Karl Fischer titration, differential scanning calorimetry, X-ray microtomography, and magnetic resonance imaging. As the principal results, matrix specific features were detected, e.g., “locking” of the internal part of the matrix (ALG/SA); existence of lamellar region associated with detection of free/freezing water (ALG/SA); existence of water penetrating the matrix forming specific region preceding infiltration layer (ALG/SNA); switch in the onset temperature of endothermic water peak associated with an increase in the fraction of non-freezing water weight per dry matrix weight in the infiltration layer (ALG/SNA). The existence of complicated spatiotemporal hydration patterns influenced by matrix composition and molecular properties of constituents has been demonstrated.

## 1. Introduction

Considering the processes taking place in polymer matrix tablets during hydration, one notices their complexity, interdependence, parallelism, and multi-faceted nature. At the same time, in the hydrating matrix, the effects related to water transport to its interior, increased rotational freedom of polymer chains, dissolution and erosion of the outer layers of the polymer matrix, polymer swelling, and the formation of viscous solutions occur. The effects of the polymer hydration are further associated with drug dissolution and diffusion effects, osmotic effects, etc. [1,2]. The result of these phenomena is responsible for macroscopic properties and the performance of the drug delivery system [3,4,5,6,7].

The studies of the phenomena occurring during the hydration of matrix tablets were initiated in the last decade of the 20th century [8,9]. The simple methods of polymer matrix expansion recording by photographing in visible light, video recording or measuring the refraction have been applied [10,11,12,13]. Starting from these studies, the existence of several fronts or regions have been proposed to describe the matrix and its temporal evolution e.g., swelling, diffusion and erosion fronts [8]; water penetration, phase transition, apparent gel, dissolution fronts [14]; penetration, swelling, erosion fronts [15] etc. The fronts terminology has been set arbitrarily with no exhaustive experimental confirmation of the actual physicochemical processes occurring therein. The studies in the proceeding decades show that the processes occurring in the hydrating matrix are much more complex [6,16].

Magnetic resonance imaging (MRI, MR imaging) has been used to study hydrophilic matrices starting from mid-90’s [17,18,19]. Spatially resolved MR relaxometry (T_2_ relaxation time mapping), one of the MR imaging techniques, has been proposed to obtain deeper insight into their molecular properties as proton distribution and mobility [20,21]. Fronts derived from optical studies have been consequently imposed on MR images or T_2_ profiles [22,23]. X-ray synchrotron microtomography [24] and X-ray microtomography (microCT) [25] have also been used for studies on the hydration of polymeric matrices in situ. Some X-ray synchrotron microtomography studies have been concentrated on freeze-dried samples [26,27]. It has been shown that the behavior of the matrix during hydration (morphology and geometrical aspects) can have an impact on the subsequent dissolution of the drug and several aspects of drug dissolution including various kinetics models, changes in dissolution kinetics and what is also important, dissolution variability [28,29]. A simple approach identifying only the external gel layer is the most common when studying hydrophilic matrices. However, at this moment, some studies allow more detailed analysis and in consequence have revealed that more complex hydration patterns are possible and are not an artifact [4,5,27]. The main challenge is to relate the matrix features observed by imaging methods with quantitative information concerning the composition of the particular matrix zones. 

In the previous study, we proposed an integrated methodology to effectively evaluate multiple spatiotemporal aspects of matrix tablet hydration: (1) quantitative water distribution using Karl Fischer (KF) titration of precisely localized samples; (2) water–polymer interaction using spatially localized differential scanning calorimetry (DSC); (3) water distribution and its molecular mobility in hydrophilic polymeric matrix upon hydration with non-invasive MRI [30]. According to the described methodology, the assessment of water content and interaction with the matrix is feasible for any area of the hydrated tablet, regardless of the hydration time and the state of the polymer–gel, sol, wetted polymer, unhydrated material, etc. In the mentioned work, placebo tablets containing sodium alginate as a matrix former have been analyzed. In the current study, we investigated matrix tablets containing the same polymer type and grade. However, the tablets also comprised active pharmaceutical ingredients (API): salicylic acid or sodium salicylate. For the purpose of the study, substances with similar molecular weight and structure which significantly differ in physicochemical properties were chosen. The solubility of salicylic acid is 2.48 g/dm^3^ and the solubility of sodium salicylate is 48.1 g/dm^3^. Both substances are often applied as model drugs [31,32,33].

The scientific hypothesis was that different and complicated hydration patterns would occur when dealing with various compositions of the sodium alginate-based matrices.

The detailed spatiotemporal characterization of the matrix during hydration could play a crucial role in the elucidation of hydration and drug dissolution mechanisms. Therefore, the goal of the study was to show that the interplay between water, tablet structure, and composition determines unique spatiotemporal hydration patterns which influence the performance of controlled release matrix tablets. The analysis of the influence of API on physicochemical properties and the behavior of tablets containing sodium alginate during hydration was carried out. Such samples under hydration are non-equilibrated, which means that the physicochemical state and morphology of the sample depend on the position in the matrix and on time. The real challenge is to take snapshots of the physicochemical properties of such a system in a reasonable time to describe its evolution. The previously described methodology, enriched by X-ray micro tomography, was applied to record and assess spatial and temporal changes of water content, its mobility and interaction with the matrix, as well as the tablet matrix morphology.

## 2. Materials and Methods

### 2.1. Materials

Sodium alginate Protanal LF 240 D was purchased from FMC Biopolymers (Philadelphia, PA, USA): the intrinsic viscosity [η] = 4.27·10^2^ cm^3^/g, average molar mass 151.955 g/mol, degree of polymerization 767, M unit content: 65–70%, G unit content: 30–35% (M/G ≈ 2). Sodium salicylate (SNA) and salicylic acid (SA) were purchased from Pharma Cosmetic (Kraków, Poland). All components were of pharmaceutical grade.

Aquametric Composite 5, methanol, and methanol high purity grade were supplied by Panreac AppliChem (Barcelona, Spain). Hydranal Composite 5 and methanol were obtained from Honeywell Fluka (Charlotte, NC, USA). All other materials used in the study were of analytical grade.

### 2.2. Tablet Preparation, Hydration and Spatial Sampling

The tablets used in the study contained a 1:1 *w*/*w* mixture of sodium alginate with salicylic acid (ALG/SA) or sodium salicylate (ALG/SNA). Round, flat matrix tablets with a diameter of 12 mm, a thickness of 6 mm and a weight of 800 mg were prepared by direct compression of powder mixtures using a single punch tablet press EK0 (Korsch-Erweka, Berlin, Germany). The hardness of all tested tablets was in the range of 40–50 N.

A spatially localized sampling of the tablet after its hydration was performed to provide spatially localized samples for Karl Fischer and differential scanning calorimetry measurements. It was feasible in the device developed especially for this purpose. It consisted of a tablet holder and a micrometric screw coupled with a piston. After unilateral tablet hydration inside the device, the tablet was cut into 1 mm thick slices by pushing up the tablet within the holder to the required position with the use of the micrometric screw. The exception was the tablet region that protruded from the holder after hydration due to polymer swelling: its sampling was not viable with the same resolution. For a detailed description of the device as well as for a mode of tablet hydration, the reader is referred to the previous study by Juszczyk et al. [30].

### 2.3. Spatial Distribution of Water Content within the Matrix Tablets Using Karl Fischer Titration

The study of water distribution in tablets during hydration by Karl Fischer titration method was carried out using 890 Titrando with Touch Control and 803 Ti Stand (Metrohm, Herrisau, Switzerland) in accordance with the experimental methodology described in Juszczyk et al. [30]. Briefly, the hydrated tablets were sampled at 1, 2, 3, and 4 h of hydration according to procedure described in Section 2.2. Samples were weighed accurately, placed into 50 mL flasks filled with methanol, sonicated for 15 min, and left for stabilization for 24 h. The water content in the samples was corrected in reference to blank measurements. The initial water content in unhydrated matrices was also determined.

### 2.4. Differential Scanning Calorimetry

The study of water distribution and its interaction with the matrix during hydration by DSC was carried out using the DSC 822e (Mettler–Toledo, Greifensee, Switzerland) with a liquid nitrogen cooling station in accordance with the experimental methodology described in Juszczyk et al. [30]. The calibration of the apparatus for temperature and enthalpy was carried out using zinc, *n*-octane, and indium standards. The samples were frozen to −80 °C at 10 °C/min, and then they were gradually heated from −80 °C to 30 °C with a scanning rate of 2 °C/min. Weight of the samples taken from the slices of hydrated tablets was in the range of 3–30 mg.

### 2.5. Magnetic Resonance Imaging and Image Analysis

MR imaging study was carried out using 9.4 T Bruker Biospin MRI scanner (Bruker, Ettlingen, Germany) and multi-slice multi-echo (MSME) pulse sequence in accordance with the experimental methodology described in details in Juszczyk et al. [30].

Briefly, the parameters of the MSME sequence were as follows: number of echoes—NE = 256, echo time—TE = 3.536 ms, repetition time—TR = 5 s, number of accumulations—NA = 1, slice thickness—1 mm, field of view—FOV = 28 × 28 mm^2^, matrix size of 256 × 256 pixels. The MSME stacks of images obtained at consecutive echo times were imported to Fiji distribution of ImageJ version 1.44 (National Institutes of Health, Bethesda, ME, USA, http://rsb.info.nih.gov/ij/, last accessed on 26 October 2021) [34]. The 14-pixel wide (1.53 mm) segment of the image was chosen. The rows of the resulting images were averaged to obtain profiles along the axis of the tablet (1D images). The pixel-by-pixel analysis of the 1D image stacks was performed to obtain 1D parametric images of T_2_ decay constant and signal amplitude A. For each profile, pixel image intensity vs. echo time was fitted using the Levenberg–Marquardt algorithm with exponential function, Equation (1).

(1) $$f\left(t\right)\;=y_{0}+Ae^{-\frac{t}{T_{2}}}$$

The calculations were made using OriginPro 2021b (OriginLab Corporation, Northampton, MA, USA).

### 2.6. X-ray Microtomography

Microtomography study was performed using Benchtop CT160 X-ray computed microtomograph (Nikon Metrology NV, Leuven, Belgium). The scanning parameters were set as follows: X-ray energy of 148 keV, current 99 µA and rotation step of 0.12°. Scanning time was optimized to 17 min per sample to be comparable with the MRI acquisition time. The 3D image volume was reconstructed using the CTpro 3D Version XT 4.4.2 (Nikon Metrology NV, Leuven, Belgium) with an isotropic voxel size of 9 μm. Further image processing was performed using Fiji distribution of ImageJ version 1.44. A 1 mm thick slice (related to 1 mm slices used in MRI study) was cropped using slice keeper stack tool. Finally, the two images were produced using the z-project stack tool: (1) image of averaged pixel intensity over all slices in 1 mm slice; (2) image containing standard deviations of pixel intensity over the slice.

### 2.7. Drug Dissolution Study

The drug dissolution study was carried out using the rotating basket method according to the European Pharmacopeia. A Hanson Vision Elite 8 dissolution tester equipped with a Hanson Vision AutoPlus Maximizer panel with a Hanson Vision AutoFill sampler and a Hanson Vision Heater was used (Teledyne Hanson Research, Chatsworth, CA, USA). The tests were carried out in distilled water for 4 h at the basket rotation speed of 100 rpm at 37 °C ± 0.5 °C. The samples were taken every 30 min. Detection of the amount of the released substances was carried out using Shimadzu UV-1800 spectrophotometer (Shimadzu USA Manufacturing Inc., Canby, OR, USA).

## 3. Results

### 3.1. Characteristics of ALG/SA Tablets

The initial water content of the unhydrated alginate tablets containing salicylic acid determined by the KF (wc_0(KF)_) method was 7.1%. After immersion of the tablet in water, an increase in total water content (wc_tot(KF)_) was observed in the whole tablet structure, regardless of hydration time. After the first hour the wc_tot(KF)_ along the axial cross-section of the ALG/SA tablet, was in the approximate range of 10–90%. In the deepest tablet slice, i.e., at the distance from the bottom of tablet *l* = 0–1 mm, the value of wc_tot(KF)_ increased from 10.1% at 1 h of hydration to 13.3% at 2 h. In the next slice (closer to the tablet surface), i.e., *l* = 1–2 mm, it raised from 12.2% to 15.8%. In the slice lying at *l* = 2–3 mm, an increase from 17% to 30% was observed, and for the region at *l* = 3–4 mm water content augmented from 41% at 1 h to 50% at 2 h. It should be noted that wc_tot(KF)_ at a distance up to *l* = 4 mm did not change much from the second to fourth hour. After 4 h the following values of wc_tot(KF)_ were determined in those slices: 12.8% (*l* between 0 and 1 mm), 16.4% (*l* between 1 and 2 mm), 32.8% (*l* between 2 and 3 mm), 51.4% (*l* between 3 and 4 mm). The data, plotted as wc_tot(KF)_ vs. time in particular slices is presented in Supplementary Materials Figure S1.

**Figure 1:**
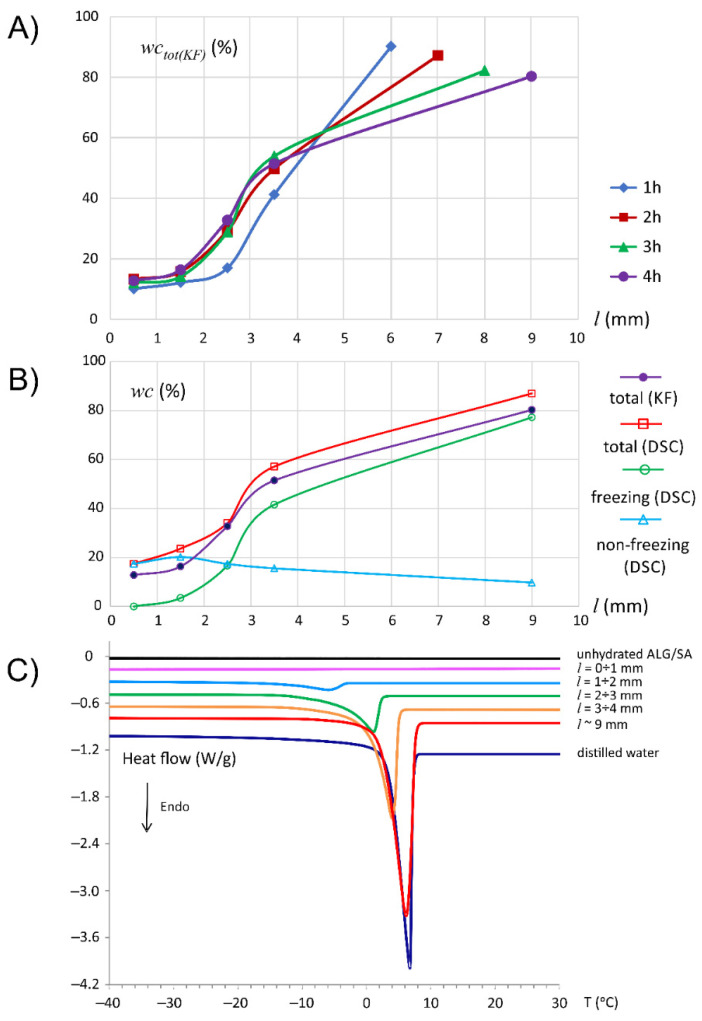
Changes in total water content (wc_tot(KF)_) in subsequent ALG/SA tablet slices at 1, 2, 3 and 4 h of hydration determined by Karl Fischer method—continuous lines serve as visual guide only (**A**); Freezing water content, non-freezing water and total water in subsequent tablet slices of the ALG/SA matrix after four hours of hydration as determined by the DSC method and total water content (wc_tot(KF)_) determined by KF method—continuous lines serve as visual guide only (**B**); DSC heating curves of the samples taken from subsequent ALG/SA matrix slices after 4 h of hydration (**C**).

The highest difference in wc_tot(KF)_ was observed between the slice *l* = 3–4 mm and the external part of the matrix, which was located in the proximity to the surrounding medium and protruded from the holder due to polymer swelling. The spatial extent of this region was dependent on the incubation time. The longer hydration, the larger the swollen area. Tablet contour was detected at varying positions, which ended up at *l* = 6 (1 h), *l* = 7 (2 h), *l* = 8 (3 h) or *l* = 9 mm (4 h). Therefore, the sample taken from this region for KF measurements corresponded to its spatial position in a particular hydration time (Figure 1A). Interestingly, the values of wc_tot(KF)_ decreased upon hydration time from 90.2% at 1 h to 80.3% at 4 h. Similar effects have been observed by Ching et al., 2008, during the hydration of systems with sodium alginate in the acidic environment [35]. The authors have linked the greater absorption of water into the alginate matrix in the early stages of hydration with the formation of cracks and gaps that arose due to local acidification of the matrix. These micro-cracks could also be formed as a result of the hydration of the ALG/SA matrix due to the presence of a weak acid (salicylic acid) in the matrix.

Basing on the Karl Fischer data, it was observed that starting from 2 h of hydration, water penetration for spatial range *l* = 0–4 mm was highly restricted and water concentration in this region was practically unchanged (see Figure 1A). The system was “locked” in terms of water penetration for this spatial range. Differences in water content at subsequent hydration times were evident for slices taken from the area above 4 mm—the evolution of the system was restricted to this region only.

The DSC studies of samples taken from particular slices of hydrated tablet at 4 h showed, that in spatial range *l* = 1–9 mm one endothermic peak corresponding to freezing water fraction in the sample was observed. On the thermogram of the slice between *l* = 0 and 1 mm (the least hydrated tablet region), no phase transition peaks were recorded. In Figure 1C the transition heat of subsequent slice peaks was normalized to sample weight. The normalized peak areas were increasing along the tablet’s axis from the tablet bottom, and it was associated with increasing content of freezing water in the sample. The onset of phase transition temperature registered for a slice between 3 and 4 mm was +1 °C, and for the external slice (*l* ≈ 9 mm): +2 °C. The thermogram of the material taken from the external slice was similar to the thermal characteristics of distilled bulk water, indicating the presence of free water in these areas. The highest absolute value of phase transition heat was recorded in the external part of the tablet (*l* ≈ 9 mm): −252 J/g, and the lowest at a slice located between 1 and 2 mm: −19 J/g. The onset of temperature transitions in the slices between 1 and 3 mm were shifted to lower temperatures (up to −12 °C), which indicated a presence of freezing bound water in the sample. The obtained results correspond to the data obtained for sodium alginate (ALG) placebo tablets [30]. Salicylic acid did not influence the formation of the additional peaks of the water phase transitions in sodium alginate tablets during its hydration (see Supplementary Materials Figure S3).

Total water content as measured by DSC was calculated as the difference of sample weight before and after its heating up to 180 °C according to the procedure described in Section 2.4. The weight of the non-freezing water fraction was calculated as a difference between total water content (wc_tot(DSC)_) and freezing water content. Figure 1B presents the individual water fractions in the samples of all tablet slices, i.e., total, freezing, and non-freezing water contents. The freezing water content in ALG/SA matrix increased from the tablet bottom towards the external part of the tablet from 0% for *l* = 0–1 mm to 77% in the external slice (*l* ≈ 9 mm). The non-freezing water content decreased from 17% in the bottom slice to about 10% in the external part of the matrix. The water present in the bottom slice was entirely a non-freezing water. In the external part, wc_tot(DSC)_ was ca. 87%.

The comparison of water content as analyzed by KF and DSC at 4 h of hydration showed the excellent agreement of the results.

In the imaging part of the study microCT and MRI were applied in separate experiments. The temporal parameters of experiments were chosen to ensure similar conditions for observations and to enable co-registration of obtained results. MicroCT fast scan was used to match reasonable temporal resolution −17 min, which was comparable with a single MRI scan. Single microCT slice was 10 μm thick and resulted in a mediocre signal to noise ratio and in consequence a low information content. Therefore, the obtained data were processed in a similar way as it was applied by Laity et al. for the processing of synchrotron microtomography data [24]. Creating averaged image over 140 slices (1.4 mm thick segment) perpendicular to the matrix axis allowed to emphasize some density related morphological features. Additionally, the standard deviation image reflected uniformity of material density in this 1 mm slice.

Magnetic resonance imaging and X-ray microtomography gave, in non-destructive manner, a more detailed overview of the hydration processes with much higher resolution than previously described results of mechanical sampling of the tablets for KF and DSC measurements.

**Figure 2:**
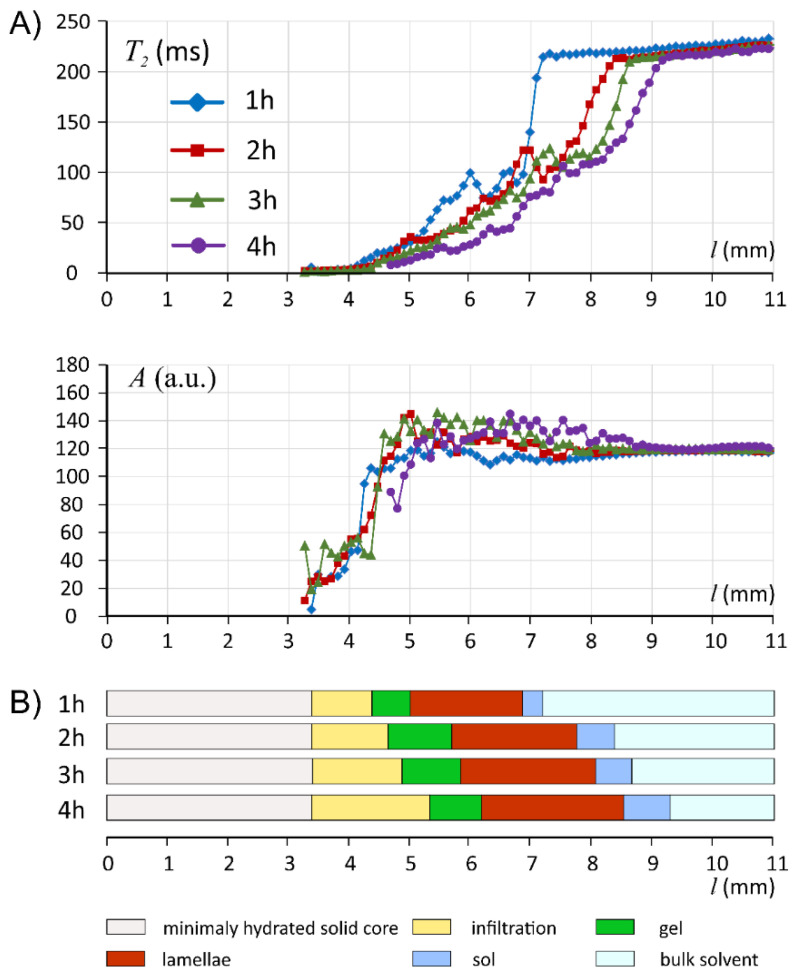
Profiles of MRI derived parameters, T_2_ relaxation time and signal amplitude (**A**), and the evolution of the interpretation of ALG/SA matrix in terms of layers at particular hydration times (**B**).

**Figure 3:**
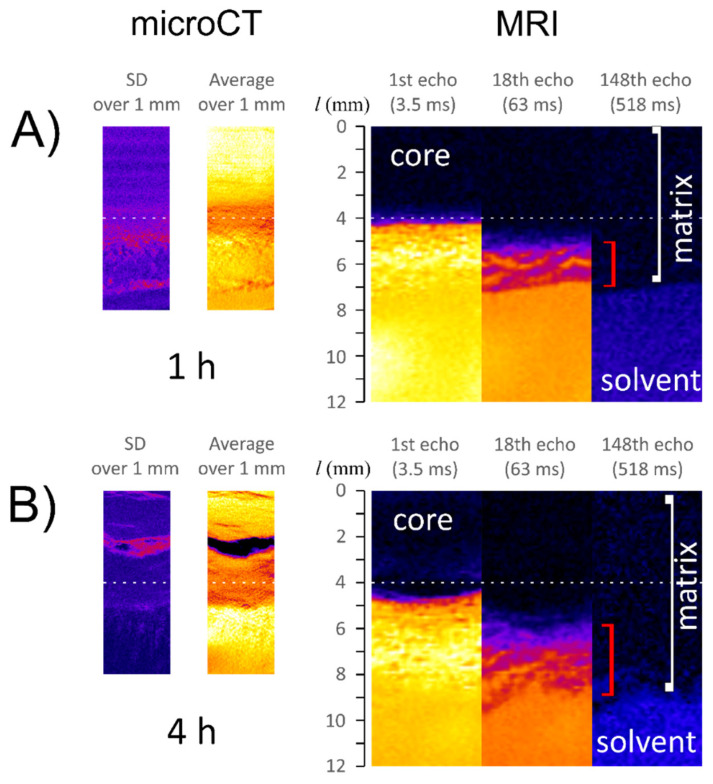
MicroCT (left) and MR images (right) of the profiles of ALG/SA matrix at 1 h (**A**) and 4 h (**B**) of hydration: microCT image containing standard deviations of pixel intensity and image of averaged pixel intensity over all slices in 1 mm thick segment; MR images of the same fragment of ALG/SA matrix at various echo times (3.5, 63 and 518 ms)—red square bracket marks lamellar region. White dashed line marks initial end-position of a dry matrix before hydration.

According to Figure 2 and Figure 3, it was apparent that the hydration pattern of ALG/SA system was different than the corresponding placebo tablet presented by Juszczyk et al. [30], i.e., there were distinct (relatively sharp) changes in T_2_/A profiles, MR images, and microCT images. When looking at spatial relaxometric profiles at 1 h of hydration, it could be noted that the part of profiles up to 4.5 mm resembled pure sodium alginate matrix, as has been presented before [30].

Starting from the matrix bottom (*l* = 0 mm) the first region, which could be identified as the core, was denoted as a minimally hydrated layer. Starting from *l* ≈ 3.5 mm at all hydration times MR signal was detected and allowed quantitative assessment of effective T_2_ relaxation time. This position marked the beginning of the infiltration layer (terminology introduced after Miller-Chou [16]) was characterized by a high gradient of MR signal amplitude (*l* in the range of 3.5–4.5 mm) for all hydration times. MicroCT cross-section obtained at 1 h of hydration showed that the region denoted in the MRI profiles as infiltration layer, corresponded to the region of lower density in a microCT image (orange zone as depicted in false color scale). This suggested matrix swelling in this layer without mass replenishment (water).

Regarding the next denoted region (gel) the starting position was identified at maximal amplitude, i.e., at the distance *l* between 4.4 and 5.4 mm. This region was relatively narrow, similar to those denoted as a gel in pure alginate, but of slightly higher T_2_ values (≈10 ms) [30]. Next, in ALG/SA T_2_ profile at 1 h of hydration spikes in T_2_ profile were observed for *l* between 5 and 7 mm, with anomalous spatial T_2_ variations at approximately constant proton density level (constant hydration level). These spikes at 4 h became less pronounced, but this region spatially expanded during hydration—at 4 h it was in the range between 6 and 9 mm. To explain this phenomenon, three MR images of the same spatial region at two representative hydration times (1 and 4 h) are presented in Figure 3. At each hydration time, 256 images (with increasing echo time) were acquired simultaneously and some of them revealed important morphological features due to different T_2_ contrast at consecutive echo times. The images presented in Figure 3 complemented T_2_/A profiles derived from MR images (Figure 2). The first echo image (TE = 3.5 ms) reflected mainly proton density and is only slightly T_1_ weighted. The image was uniform concerning image intensity, and it was only slightly grainy, which was an effect of possible micropores or microbubbles at sub-pixel level i.e., <100 μm. MicroCT images confirmed the existence of micropores and microbubbles. The image of the same fragment obtained at the echo time of 63 ms revealed morphological details of the matrix inside the 5–7 mm region (at 1 h of hydration). Despite the almost constant proton density in this region, the matrix was heterogeneous on a macro scale in terms of local spatial variations of T_2_. These variations formed irregular ca. 300 μm thick lamellae oriented almost perpendicularly to the matrix axis. The heterogeneity of this region was at the molecular level, it reflected water molecular dynamics. The only spatial variations were observed in T_2_. MRI derived proton density or matrix density according to microCT results were homogeneous. One can hypothesize concerning the origin of this region, which is absent in the pure sodium alginate matrix, that the slight acidification of the matrix caused by dissolving salicylic acid led to the modification of the internal gel structure. This observation corresponded with the decrease in water content in the external layer of the matrix at subsequent hydration times as measured by KF. The results of MRI relaxometry were related to spatially resolved DSC measurements obtained at 4 h of hydration. At ca. 9 mm, i.e., in a lamellar region, the bulk water was observed.

Following the lamellar region, a very narrow zone with steeply increasing T_2_ could be observed (denoted as sol). At the echo time of 518 ms, only pure solvent was visible as a blue region. At such long echo time, the signal from the matrix was attenuated, giving a clear matrix/solvent border.

In the external region containing medium slight T_2_ drop from ≈220 ms at 1 h down to ≈210 ms at 4 h of hydration was observed in subsequent hydration times. This implies marginal erosion of the system in terms of matrix dissolution.

According to dissolution profiles presented in Supplementary Materials Figure S4, partial release of salicylic acid was observed. Therefore, it could be postulated that phenomena observed in the ALG/SA matrix, i.e., (1) the “squeezing” of water from the external layer of the matrix; (2) the formation of lamellar structures in the middle, which (3) led to drastic “locking” of the internal part of the matrix system, were caused by the changes of polymer solubility, connected with the reversal of dissociation of alginate chains in acidic environment created by dissolving SA. In the placebo alginate matrix, such a drastic “locking” of the internal part of the matrix has not been observed. The total water content in ALG matrix for *l* = 0–4 mm slightly but steadily increased [30].

### 3.2. Characteristics of ALG/SNA Tablets

**Figure 4:**
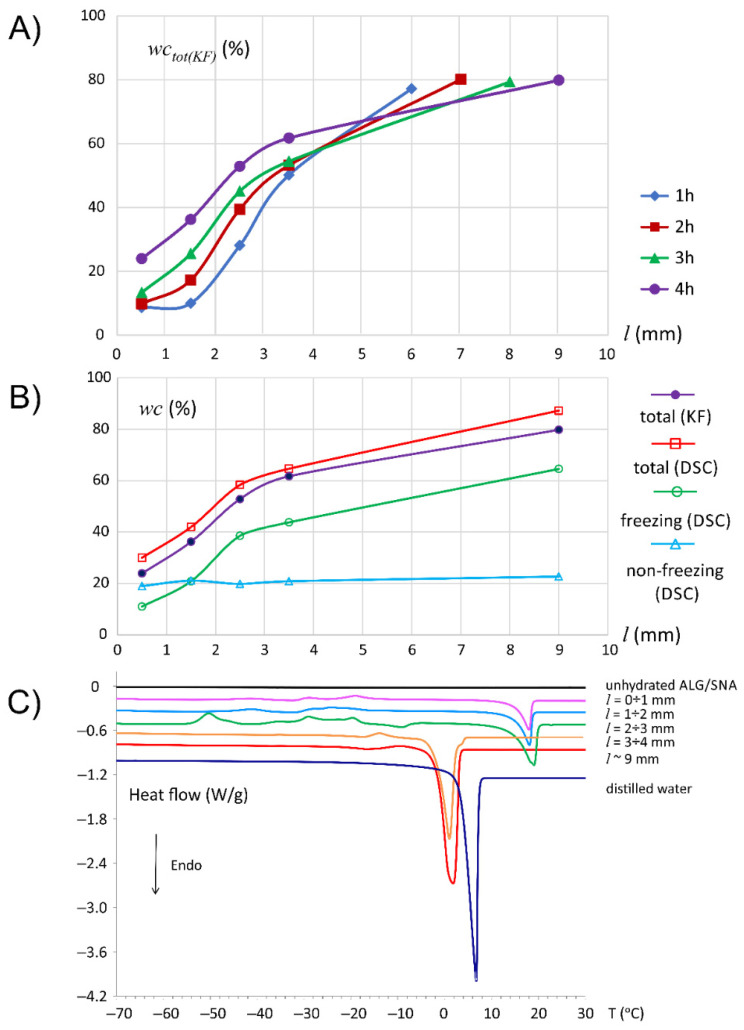
Changes in total water content (wc_tot(KF)_) in subsequent ALG/SNA tablet slices at 1, 2, 3 and 4 h of hydration determined by Karl Fischer method—continuous lines serve as visual guide only (**A**); Freezing water content, non-freezing water and total water in tablet slices of the ALG/SNA matrix after four hours of hydration as determined by the DSC method; and total water content (wc_tot(KF)_) determined by KF method—continuous lines serve as visual guide only (**B**); DSC heating curves of samples taken from subsequent ALG/SNA slices after 4 h of hydration (**C**).

The initial water content of the unhydrated alginate tablets containing SNA determined by the KF method was 7.7%, a situation similar to ALG/SA matrix. The content of water absorbed in the slices of the ALG/SNA matrix from the bottom to *l* = 4 mm gradually increased with time (see Figure 4A). After the first hour of hydration, the slice located at the tablet bottom (*l* = 0–1 mm) contained 8.6% of water. During the next three hours, it increased, up to 23.9%. Initially, a small amount of water was determined between 1 and 2 mm (10.0%), and at the end of the experiment, it reached 36.2%. The next slice contained, at distance *l* between 2 and 3 mm, from 28.1% to 52.9% of water in subsequent hours. For *l* = 3–4 mm a 50.2% water content was observed after 1 h of hydration. It increased up to 61.7% after 4 h. The total water content of the external, swollen part of the matrix did not change noticeably. After the first hour of hydration wc_tot(KF)_ of the external part (*l* = 6 mm) was 77.2% and in the following hydration hours it was about 80% (*l* = 7 mm for 2 h, *l* = 8 mm for 3 h or *l* = 9 mm for 4 h). The plot wc_tot(KF)_ vs. time in particular slices are presented in Supplementary Materials Figure S2. The presence of a freely soluble sodium salicylate increased the penetration of water into the system when compared with ALG/SA and placebo ALG matrix [30]. Similar observations are reported in the literature concerning other hydrophilic polymer matrices [36,37]. The obtained results confirmed the influence of API of different physicochemical properties on the content of absorbed water in individual regions of sodium alginate-based matrices. The characteristics of the ALG/SNA matrix thermograms obtained after 4 h of hydration (Figure 4C) differed from the results obtained for ALG/SA in the number of peaks observed and their position. For *l* in the range from 0 to 3 mm, the onset of an endothermic water peak was found at a temperature of +15 °C. At temperatures lower than −5 °C, there were identified some exothermic or exo-endothermic phase transitions with very low values of the phase transition heat of 10 J/g or lower.

The thermal properties of ALG/SNA tablet slices were related to those of SNA/water solution (See Supplementary Materials Figure S3). In both cases, onsets of exothermic peaks were observed at temperatures below −8 °C and one endothermic peak at approx. +15 °C. It indicated a dominant influence of sodium salicylate interaction with water on the properties of the matrix in the deeper regions of the tablet, with respect to water-polymer interactions. The phase transition heat of the endothermic peak of the slices from 0 to 3 mm at ca. +15 °C ranged from −115 J/g to −48 J/g, and their normalized values increased towards the surrounding medium which translated into (increasing content of freezing water).

When moving further towards the external part of the matrix a drastic change in the onset position of endothermic water peak was observed for these regions (*l* > 3). In the matrix regions *l* = 3–4 mm and the external part of the matrix (*l* ≈ 9 mm), the onset of the endothermic peak was observed at −3 °C. The heat of phase transition was −84 J/g for *l* = 3–4 mm, and for the external part of the matrix, it was −205 J/g. The additional exothermic peak was recorded at −17 °C and in the external part at −22 °C.

The freezing water content in ALG/SNA tablet at the 4 h increased from the bottom towards the external part of the tablet from 11% to 65% (Figure 4B). The content of non-freezing water remained at the level of about 20% along the whole matrix. Total water content increased towards the medium achieving 87% at *l* ≈ 9 mm. In the ALG/SNA thermogram shown in Figure 4C, there were exothermic and endothermic peaks at temperatures lower than −5 °C. Their presence did not necessarily mean the existence of water with different properties. According to Hofer et al. and Kyritsis et al. [38,39] the existence of numerous peaks in the DSC thermograms of some polymers may be a consequence of crystallization, melting, and re-crystallization of the same water fraction. Therefore, the values of freezing and non-freezing water contents for ALG/SNA determined in this DSC study should be considered as approximated. An additional difficulty in the interpretation of thermal behavior of the systems composed of sodium alginate, sodium salicylate and water was connected with hydrotropic properties of SNA [40]. Due to the fact that the hydrotropism takes place beyond a certain critical concentration called hydrotropic concentration (MHC) and the concentrations of SNA differ within the matrix, the interpretation of this phase transitions was not possible without additional studies.

**Figure 5:**
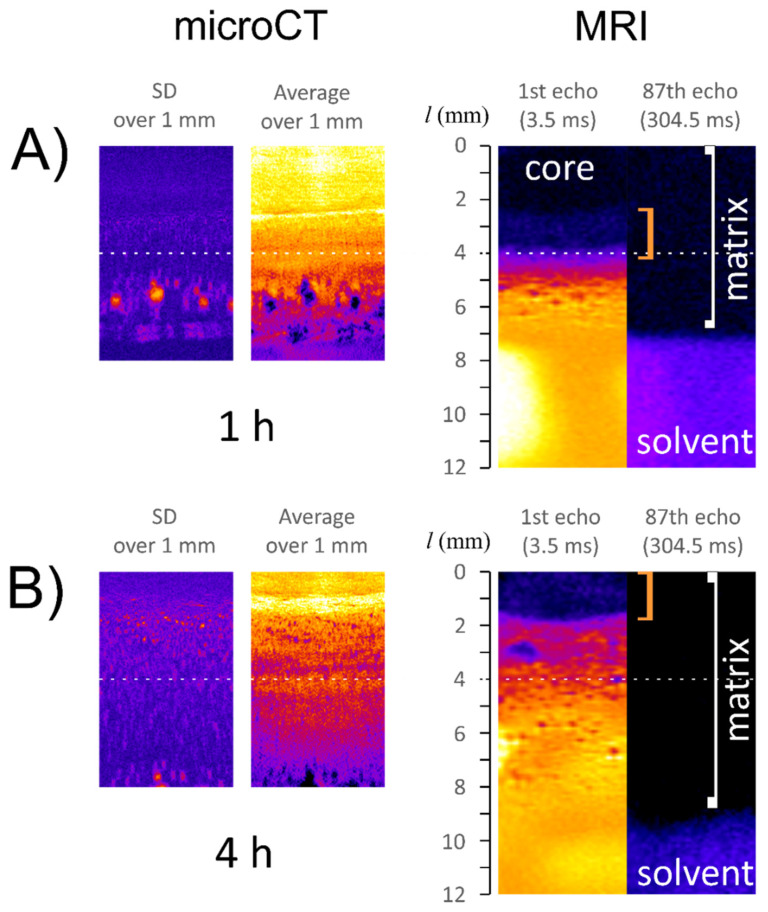
MicroCT (left) and MR images (right) of the profiles of ALG/SNA matrix at 1 h (**A**) and 4 h (**B**) of hydration: microCT image containing standard deviations of pixel intensity and image of averaged pixel intensity over all slices in 1 mm thick segment; MR images of the same fragment of ALG/SNA matrix at two echo times (3.5 and 304.5 ms)—orange square bracket marks region denoted as swollen-glassy as introduced by Ju et al. [41]. White dashed line marks initial end-position of the dry matrix before hydration.

**Figure 6:**
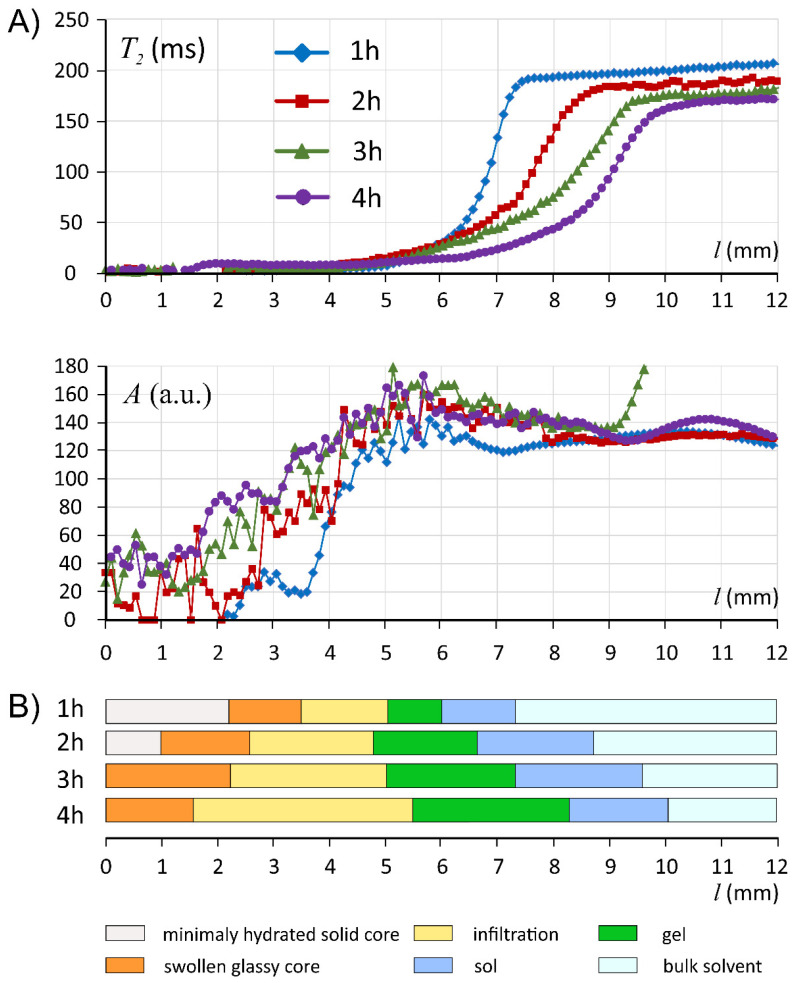
Profiles of MRI derived parameters, T_2_ relaxation time and signal amplitude (**A**) and the interpretation of evolution of the ALG/SNA matrix in terms of layers at particular hydration times (**B**).

In the case of ALG/SNA tablets, the minimally hydrated solid core was observed using MRI only at 1 and 2 h of hydration. Two images of the same spatial region are presented in Figure 5 at representative hydration times (1 h and 4 h). It was evident that the ALG/SNA matrix promoted water penetration in contrast to ALG/SA—the dark blue region marked by orange bracket between core and infiltration layer was clearly visible. In T_2_/A profiles (Figure 6) this region was depicted as a distinct “step” in amplitude profile at *l* ≈ 2.5 mm at 1 h of hydration. A similar region of uniform proton density preceding high proton density gradient has been observed in HPMC based quetiapine fumarate matrices. Such a region according to previous works by Ju et al. has been denoted as swollen-glassy [29,41,42,43,44]. With the beginning of the “step” as seen in MR images, a distinctly bright and narrow front was associated with microCT images, suggesting a higher density of the material. A similar front has been observed by Laity et al. when using synchrotron X-ray microtomography for studying HPMC/MCC matrix [24].

Unlike ALG/SA, Karl Fischer results suggest moving of hydration front towards the bottom of the matrix (i.e., towards *l* = 0), which was characterized by increasing water content above non-freezing content. It was also reflected in the MRI amplitude profile: the moving front was observed at the beginning of the infiltration layer. The infiltration layer was relatively narrow at 1 h of hydration. Then, at subsequent hydration times, it became broader and, in consequence, less steep. The corresponding T_2_ relaxation times in this layer were at the detection limit (i.e., lower than 10 ms). Interestingly, the end-position of the infiltration layer at all studied hydration times (1–4 h) was at about 4.5 mm and was synchronized with a simultaneous increase in T_2_ profiles. According to KF at 4 h, the “orange bracket” region was related to the appearance of the freezing water pool in the matrix. Between 2.5 mm and 3.5 mm, i.e., somewhere at half of the slope of infiltration layer, water phase transition onset temperature was switched from ca. 0 °C to ca. −10 °C, which indicated the presence of freezing bound water in that region

Starting from the end of the infiltration layer (or beginning of fully hydrated zone), T_2_ increased spatially towards the medium, which was characterized by T_2_ plateau. The change in the character of T_2_ increase was evident at 2 and 3 h of hydration (*l* = 7.4 mm and *l* = 7.7 mm, respectively). Such a switch-point was practically absent at 1 h. At 4 h, it was evident at about 8.3 mm but less pronounced. The switch-point marks additional “front” dividing the fully hydrated part of the matrix into two separate layers (gel and sol). In studies on HPMC matrices such a switch-point has been observed and interpreted as related to drug substance dissolution, as it was absent in HPMC placebo matrix [4,5]. Microbubbles were clearly visible in microCT at 1 h for *l* = 5–6 mm and they were also detected in MR image on the first echo, as spots of lower intensity. It implied that the consistency of the matrix allowed the formation of spherical air bubbles instead of cracks which is a very important observation. Swelling of ALG/SNA matrix was slightly more effective. At 4 h the tablet border was at ≈10 mm, while for ALG/SA matrix it was 9 mm. In this case, the whole volume of the matrix was hydrated and evolved, while for ALG/SA matrix only part of the matrix (*l* > 3.5) effectively expanded under hydration.

In the bulk solvent region (*l* > 7 mm at 1 h, *l* > 9 mm at 2 h, *l* > 9.5 mm at 3 h *l* > 10 mm at 4 h) in subsequent hydration times substantial drop from ≈200 ms at 1 h down to ≈170 ms at 4 h of hydration was observed. This indicated that dissolved matrix products were present in the dissolution medium lowering T_2_ relaxation time.

### 3.3. Discussion with Reference to Alginate (Placebo) Matrix

**Figure 7:**
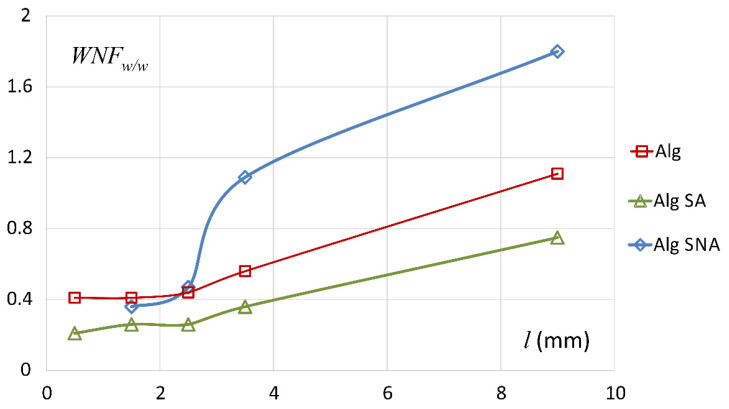
The fraction of non-freezing water weight per dry matrix weight expressed as WNF_w/w_ (detailed description in the text) for subsequent slices of ALG/SA, ALG/SNA and ALG matrices after hydration in distilled water for 4 h—continuous lines serve as visual guide only.

Figure 7 is a good starting point for a discussion on the differences between the alginate matrix systems and it is crucial for understanding the differences between them. It presents the spatial distribution of the non-freezing water weight to dry matrix weight ratio (WNF_w/w_) for ALG (placebo matrix), ALG/SA and ALG/SNA matrices after four hours of hydration in water. Data for placebo sodium alginate matrix are presented as a reference and they are taken from the work by Juszczyk et al. [30]. The WNF_w/w_ ratio shows the interaction between water and polymer/API binding sites and its value does not directly depend on the total water content in the sample. ALG/SA matrix was characterized by a relatively constant level of WNF_w/w_ in the slices from *l* = 0 to *l* = 3 mm. Its values were of ca. 0.21–0.26, i.e., half the value obtained for ALG, which can be explained by lower polymer content in the matrix—in the ALG/SA matrix the polymer constituted only half of the total matrix mass. In subsequent slices of ALG/SA, the parameter increased to 0.36 (*l* between 3 and 4 mm) and in the external part it achieved the value of 0.75. It is worth mentioning that the values of WNF_w/w_ for ALG/SA matrix were in each slice lower than for the ALG placebo matrix as presented in the work by Juszczyk et al. [30]. Despite this, both matrices had a similar thermal character as seen by DSC. The obtained results indicated a similar mechanism of interaction of the polymer with water.

In the slices situated between *l* = 1 mm and *l* = 3 mm in ALG/SNA matrix, the values of WNF_w/w_ were 0.36–0.44. They were similar to placebo tablets (ALG) presented in the previous study [30]. In the slice between *l* = 3 and *l* = 4 mm WNF_w/w_ was 1.09. A sharp spatial increase was observed between layer 2–3 mm and 3–4 mm. It must be pointed out that the content of non-freezing water in the matrix was maintained on the constant level ca. 20%, as can be seen in Figure 4.

Such a sharp spatial increase in WNF_w/w_ was spatially co-registered with changes in the onset temperature values of phase transition of water present in tablet regions between the slices 2–3 mm and 3–4 mm. These onset temperatures changed from ca. 10 °C down to ca. −6 °C in those regions. The content of non-freezing water was approximately constant across the matrix, ca. 20%, while in ALG/SA as well as in pure ALG it was decreasing towards the medium. T_2_ step appeared at about 2 mm (10 ms) and it was retained up to *l* ≈ 5 mm—all described above changes occurred at a relatively low molecular mobility of non-freezing water of 10 ms (region denoted as infiltration layer—at 4 h stretched). The ratio WNF_w/w_ was 1.80 in case of the external tablet area (*l* = 9 mm), which was the highest recorded value among the tested tablets. The presence of SNA, the easily soluble drug, in the matrix changed the properties of the system when comparing with ALG/SA and ALG ALG (placebo tablet). After its dissociation, salicylate ions caused the repulsion of polymer chains as both compounds had negative charges [45]. This could favor the formation of spaces that might serve as a reservoir for water molecules in non-freezing state.

**Figure 8:**
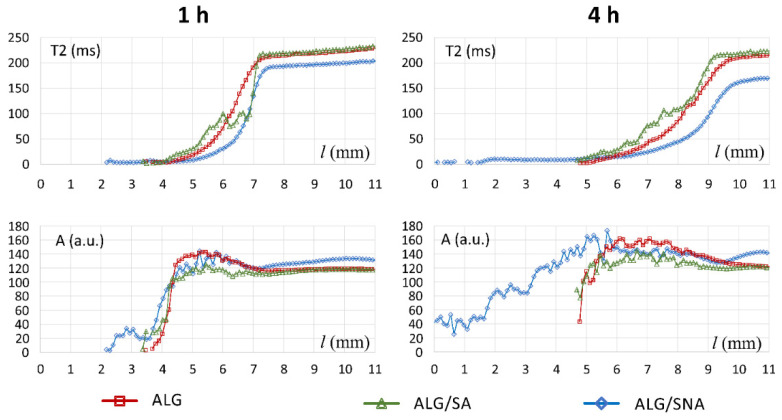
Comparison of T_2_/A profiles for three different matrices: ALG, ALG/SA, ALG/SNA at two hydration times of 1 and 4 h.

To emphasize the differences between the ALG/SA and ALG/SNA Figure 8 presents their MRI derived profiles of T_2_ and corresponding signal amplitude at 1 and 4 h in a single diagram together with profiles of placebo alginate matrix (ALG). The results for ALG have been described in details in the previous work by Juszczyk et al. [30] and serve as an introduction to more complicated cases, i.e., matrices with the addition of drugs ALG/SA and ALG/SNA. It should be noted that initial water content in ALG/SA and ALG/SNA matrices (7.1% and 7.7%, respectively) as a measure by KF was about half of the initial water content as measured in pure alginate matrices [30], while the polymer content in ALG/SA and ALG/SNA matrices was 50% *w/w*.

When comparing T_2_ profiles obtained at 1 h of hydration, it was apparent that the T_2_ values in ALG/SA for spatial locations inside the matrix for *l* above 4 mm were higher than for ALG/SNA. They were in the range ≈50–100 ms for ALG/SA (*l* = 5.3–6.7 mm), while T_2_ relaxation times for *l* < 6.5 mm of ALG/SNA were lower than 50 ms. This implied lower mobility of water in the fully hydrated part of ALG/SNA matrix. A “step” in amplitude profile of ALG/SNA at ca. 2.5 mm at 1 h of hydration did not exist in ALG [30] or ALG/SA or it was very narrow. The bulk solvent region in the ALG/SNA region (*l* above 7.5 mm at 1 h, or above 10 mm at 4 h) had lower T_2_ relaxation times than pure ALG and ALG/SA matrices. This suggested that in the ALG/SNA matrix the internal environment facilitated the disentangling of polymeric chains, which in macro-scale resulted in the increase of matrix viscosity and on a molecular level the decrease of proton mobility. This implied dissolution of the matrix and the formation of a viscous solution containing products of matrix dissolution outside of the matrix. Moreover, the heterogeneous region containing lamellae in ALG/SA was specific for this matrix composition (formulation). Indeed, its structure evolution suggested possible further erosion of the system in terms of sequestration rather than dissolution during incubation times longer than 4 h.

## 4. Conclusions

The study presents the first attempt to co-register spatially the results of several spatially resolved methods including two nondestructive imaging methods, i.e., relaxometric MR imaging together with X-ray microtomography and two destructive Karl Fischer and DSC to observe hydration patterns in two alginate-based matrix systems. It allowed identifying of specific hydration patterns for each matrix composition. When dealing with various compositions, complicated hydration patterns can be expected. In this particular case, various hydration patterns were obtained for alginate with salicylic acid (ALG/SA) and alginate with sodium salicylate (ALG/SNA).

The presented hybrid destructive-nondestructive methodology was a link between well-established methods (non-spatially resolved) and spatially resolved analysis using magnetic resonance imaging and X-ray microtomography. For the first time, four different methods were spatially co-registered (i.e., Karl Fischer titration, differential scanning calorimetry, X-ray microtomography, and magnetic resonance imaging) to provide a complex picture of polymeric matrix hydration. Hydrated alginate-based matrices generate varied hydration patterns which cannot be described simply by one or two hydration layer models. The existence of up to five different regions (layers) was identified in studied alginate-based matrices. An attempt to the interpretation of the hydration patterns was made according to the results of spatially resolved magnetic resonance relaxometry in terms of T_2_ relaxation time and decay envelope amplitude (roughly the proton density) and the results of differential scanning calorimetry.

The results of the study showed that it was hard to generalize concepts of hydration of polymeric matrices, especially when dealing with polyelectrolytes. Such generalizations did not work. The existence of complicated spatial morphological/molecular hydration patterns are demonstrated by several features, including:

- “locking” of the internal part of the matrix (ALG/SA);
- existence of lamellar region associated with detection of free/freezing water (ALG/SA);
- existence of water penetrating the matrix, forming a specific layer preceding the infiltration layer (ALG/SNA);
- switch in the onset temperature of endothermic water peak associated with an increase in the fraction of non-freezing water weight per dry matrix weight in the middle of infiltration layer (ALG/SNA);
- the region denoted as infiltration layer is characterized by a high gradient in magnetic resonance signal intensity (proton density) and lower X-ray absorbance (mainly lower density) when compared with the core.

The existence of such specific patterns suggests the need for a revision of existing approaches to matrix hydration (e.g., in terms of hydration, swelling and diffusion fronts).

## Data Availability

The processed data required to reproduce these findings is available from the authors upon reasonable request.

## Supplementary Materials

The following are available online at https://www.mdpi.com/article/10.3390/ma14216531/s1, Figure S1: Changes in total water content (wc_tot(KF)_) in subsequent ALG/SA tablet slices at 1, 2, 3 and 4 h of hydration determined by Karl Fischer method—an alternative presentation: wc_tot(KF)_ vs. time, Figure S2: Changes in total water content (wc_tot(KF)_) in subsequent ALG/SNA tablet slices at 1, 2, 3 and 4 h of hydration determined by Karl-Fischer method—an alternative presentation: wc_tot(KF)_ vs. time., Figure S3: DSC heating curves of drug (salicylic acid, sodium salicylate) solutions in H_2_O. Water heating curve is included for reference purpose, Figure S4: Dissolution profiles of sodium salicylate and salicylic acid from ALG/SA and ALG/SNA matrices respectively (*n* = 3).
