# Supplementary material for: Hydration Patterns in Sodium Alginate Polymeric Matrix Tablets—The Result of Drug Substance Incorporation
Source: Materials (Basel). 2021 Oct 29;14(21):6531. doi: 10.3390/ma14216531 (PMC8585188; doi:10.3390/ma14216531)
Supplement: Supplementary file 1 [file materials-14-06531-s001.zip › materials-1421464-supplementary.pdf]

materials

Supplementary Materials

Hydration Patterns in Sodium Alginate Polymeric Matrix Tablets—The Result of Drug Substance Incorporation

Ewelina Juszczyk [1], Piotr Kulinowski [2,*], Ewelina Baran [2], Artur Birczyński [2], Jolanta Klaja [3], Dorota Majda [4], Encarna Garcia-Montoya [5], Władysław P. Węglarz [6] and Przemysław Dorożyński [7]

[1] Research and Development Center, Celon Pharma S.A., Marymoncka 15, 05-152 Kazuń Nowy, Poland; ewelina.juszczyk@celonpharma.com
[2] Institute of Technology, The Pedagogical University of Kraków, Podchorążych 2, 30-084 Kraków, Poland; ewelina.baran@up.krakow.pl (E.B.); artur.birczynski@up.krakow.pl (A.B.)
[3] Oil and Gas Institute—National Research Institute, Lubicz 25 A, 31-503 Kraków, Poland; klaja@inig.pl
[4] Faculty of Chemistry, Jagiellonian University, Gronostajowa 2, 30-387 Kraków, Poland; majda@chemia.uj.edu.pl
[5] Pharmaceutical Technology and Physico-Chemical Department, Universidad de Barcelona, Av. Joan XXIII 27-31, 08028 Barcelona, Spain; encarnagarcia@ub.edu
[6] Department of Magnetic Resonance Imaging, Institute of Nuclear Physics Polish Academy of Sciences, Radzikowskiego 152, 31-342 Kraków, Poland; wladyslaw.weglarz@ifj.edu.pl
[7] Department of Drug Technology and Pharmaceutical Biotechnology, Medical University of Warsaw, Banacha 1, 02-097 Warszawa, Poland; przemyslaw.dorozynski@wum.edu.pl
* Correspondence: piotr.kulinowski@up.krakow.pl

Supplementary Materials

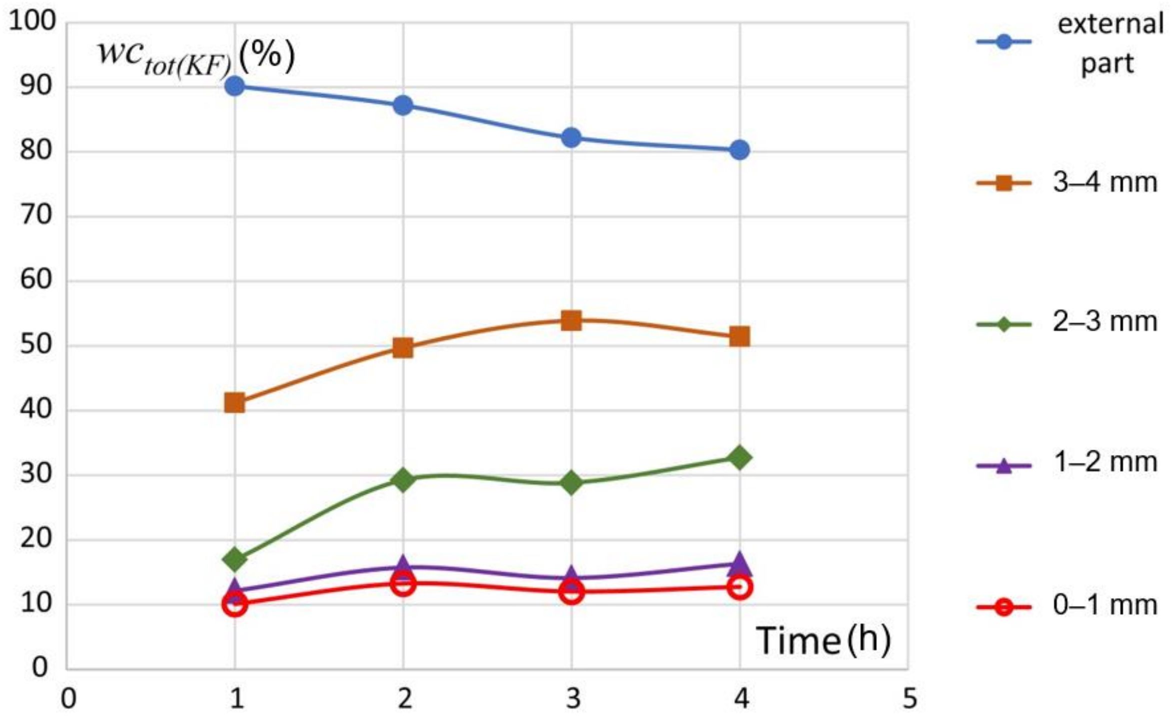

Figure S1. Changes in total water content ($wc_{tot(KF)}$) in subsequent ALG/SA tablet slices at 1, 2, 3 and 4 h of hydration determined by Karl Fischer method—an alternative presentation: $wc_{tot(KF)}$ vs. time.

Position of external part varied depending hydration time: *l* c.a. 6 at 1 h, *l* c.a. 7 at 2 h, *l* c.a. 8 at 3 h, *l* c.a. 9 at 4 h.

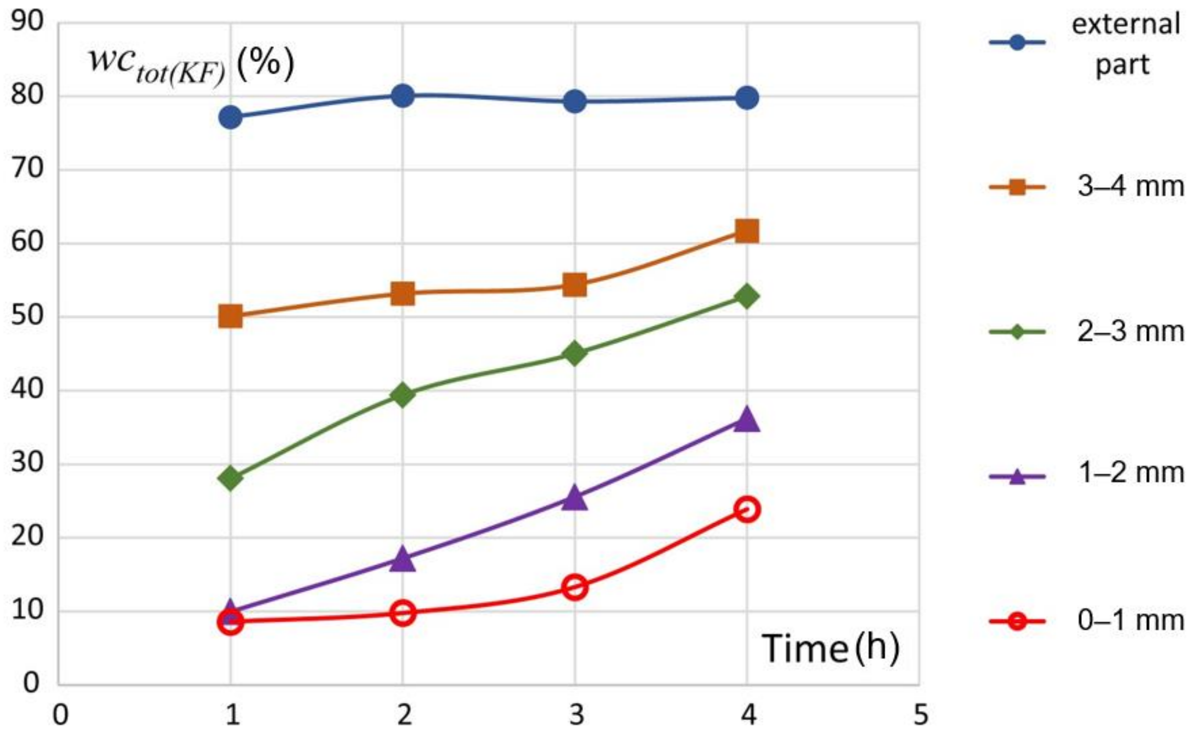

Figure S2. Changes in total water content ($wc_{tot(KF)}$) in subsequent ALG/SNA tablet slices at 1, 2, 3 and 4 h of hydration determined by Karl-Fischer method—an alternative presentation: $wc_{tot(KF)}$ vs. time.

Position of external part varied depending hydration time: *l* c.a. 6 at 1 h, *l* c.a. 7 at 2 h, *l* c.a. 8 at 3 h, *l* c.a. 9 at 4 h.

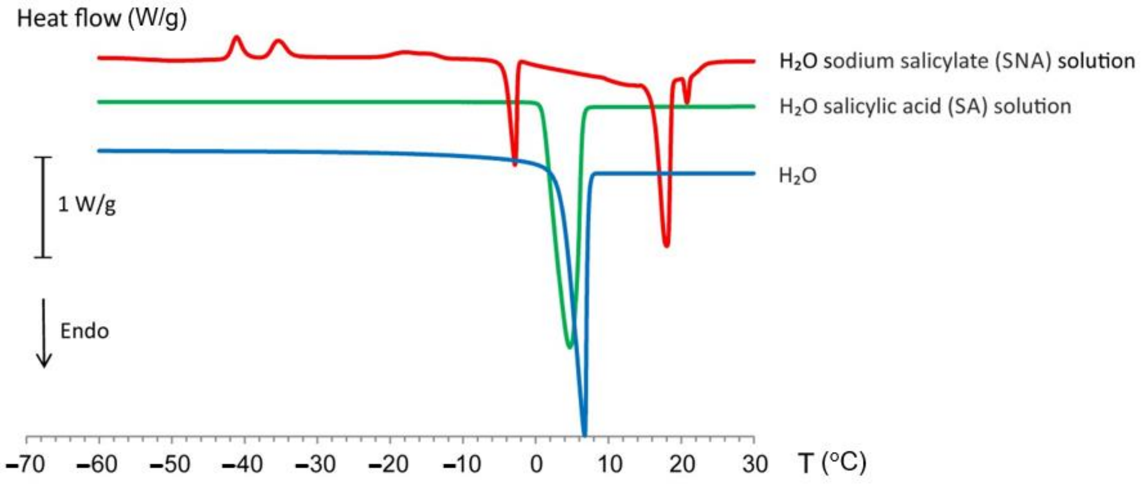

Figure S3. DSC heating curves of drug (salicylic acid, sodium salicylate) solutions in H_2O. Waterheating curve is included for reference purpose.

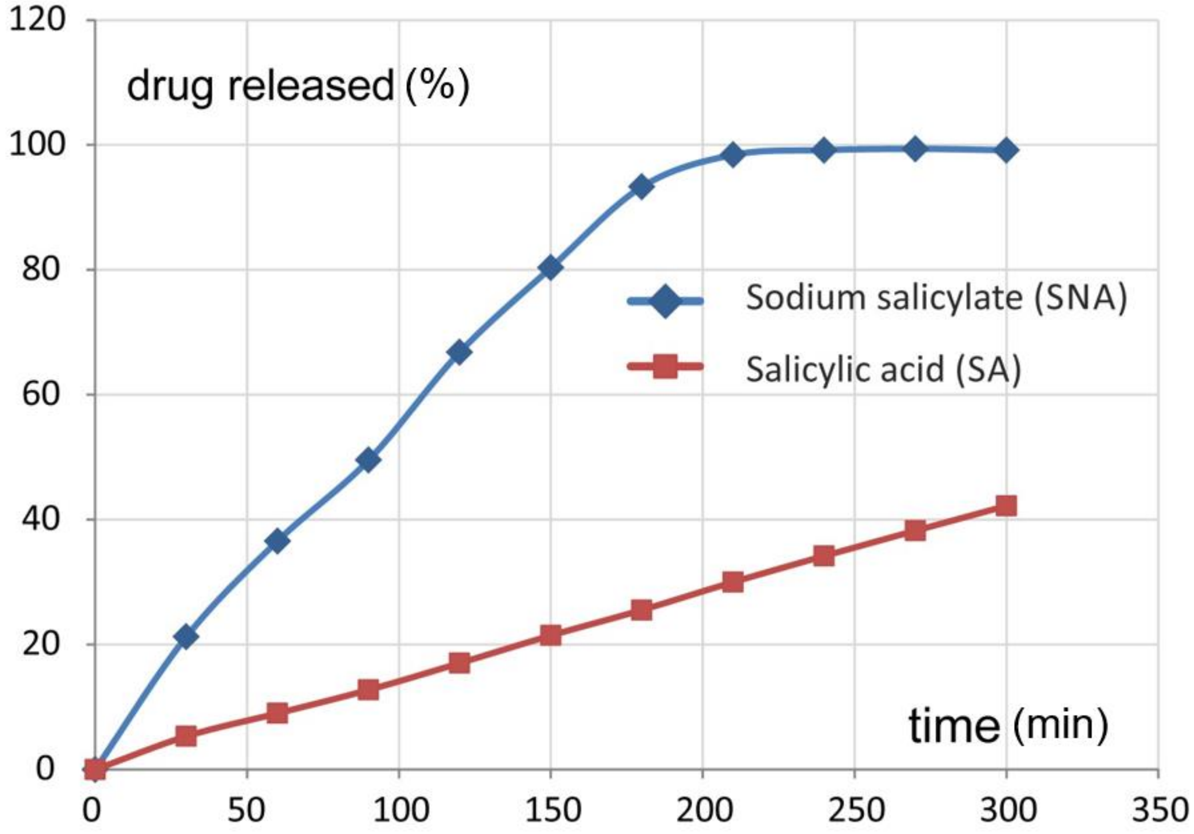

Figure S4. Dissolution profiles of sodium salicylate and salicylic acid from ALG/SA and ALG/SNA matrices respectively (n = 3).

In the case of the ALG/SA matrix, a linear relationship between the amount of the released substance and the release time (R2 = 0.9992) was observed over the entire time interval of the experiment. After the first hour, 9% of salicylic acid was released, and after four hours—34%. Under the same experimental conditions, more drug substance was released from the ALG/SNAmatrix than from the ALG/SA matrix at each sampling point. After the first hour, 37% of sodiumsalicylate had passed into the solution, and its complete release was observed at the end of the study. The complete dissolution of sodium salicylate after 4 hours and partial dissolution of salicylic acid are consistent with the observed hydration behavior of these two polymeric matrix systems.
